# SY-Net: A Rice Seed Instance Segmentation Method Based on a Six-Layer Feature Fusion Network and a Parallel Prediction Head Structure

**DOI:** 10.3390/s23136194

**Published:** 2023-07-06

**Authors:** Sheng Ye, Weihua Liu, Shan Zeng, Guiju Wu, Liangyan Chen, Huaqing Lai, Zi Yan

**Affiliations:** 1 School of Electric & Electronic Engineering, Wuhan Polytechnic University, Wuhan 430023, China; yesheng19982023@163.com (S.Y.); chenliangyan@whpu.edu.cn (L.C.); lhq15527756213@163.com (H.L.); yz9915@163.com (Z.Y.); 2 School of Mathematics & Computer Science, Wuhan Polytechnic University, Wuhan 430023, China; zengshan1981@whpu.edu.cn; 3 The Key Laboratory of Earthquake Geodesy, Institute of Seismology, China Earthquake Administration, Wuhan 430023, China; wuguiju@eqhb.gov.cn

**Keywords:** instance segmentation, deep learning, rice seed, small target, feature fusion

## Abstract

During the rice quality testing process, the precise segmentation and extraction of grain pixels is a key technique for accurately determining the quality of each seed. Due to the similar physical characteristics, small particles and dense distributions of rice seeds, properly analysing rice is a difficult problem in the field of target segmentation. In this paper, a network called SY-net, which consists of a feature extractor module, a feature pyramid fusion module, a prediction head module and a prototype mask generation module, is proposed for rice seed instance segmentation. In the feature extraction module, a transformer backbone is used to improve the ability of the network to learn rice seed features; in the pyramid fusion module and the prediction head module, a six-layer feature fusion network and a parallel prediction head structure are employed to enhance the utilization of feature information; and in the prototype mask generation module, a large feature map is used to generate high-quality masks. Training and testing were performed on two public datasets and one private rice seed dataset. The results showed that SY-net achieved a mean average precision (mAP) of 90.71% for the private rice seed dataset and an average precision (AP) of 16.5% with small targets in COCO2017. The network improved the efficiency of rice seed segmentation and showed excellent application prospects in performing rice seed quality testing.

## 1. Introduction

Rice is one of the most important staple foods in the world. The price of rice seeds varies greatly depending on variety, origin and quality, so the quality of rice seeds needs to be measured during marketing and processing. The traditional rice seed quality inspection approach is performed manually by experts, and it is time-consuming and easily influenced by subjective factors. In recent years, nondestructive rice seed inspection technology based on optical image processing technology has received widespread attention. Accurately segmenting rice seeds with similar physical characteristics, small particles and dense distributions from their backgrounds is critical for accurately determining the quality of rice seeds. Two types of traditional image segmentation methods are available: threshold-based methods and graph theory-based methods. Threshold-based segmentation methods calculate one or more thresholds based on the greyscale features of an image and assign pixels to appropriate classes based on these thresholds. Typical methods include adaptive thresholding [1], maximum entropy thresholding [2] and OSTU thresholding [3], but these methods use only the greyscale information from a given image, which inevitably causes them to misclassify the noise in the background and thus affects their accuracy. Graph theory-based methods solve the segmentation problem by optimizing an objective function. Typical methods include the one-cut [4], normalized cut [5], min–max cut [6] and graph cut [7] approaches. These methods are insensitive to the shape of the target and are too computationally intensive to be suitable for rice seed segmentation.

In recent years, hyperspectral detection technology [8,9,10,11,12,13] has achieved good results in target segmentation and quality detection tasks by simultaneously using image information and spectral information from seeds. For example, in 2020, S. D. Fabiyi et al. [14] combined hyperspectral image data and high-resolution RGB image data to segment rice seeds and more accurately acquire their spatial and spectral information to determine their categories, achieving a maximum recognition rate of 98.59% across six rice species. In 2021, Liu et al. [15] used hyperspectral techniques to segment and classify four rice seed images with an average recognition rate of 98%. Jun Zhang et al. [16] used hyperspectral techniques to obtain the spatial and spectral information for maize seeds with different degrees of frostbite, selected the best segmentation wavelength for the maize seeds and identified the degree of frostbite exhibited by them based on the segmentation information, achieving an accuracy of 97.5% corresponding to five degrees of frostbite. Although performing image segmentation with hyperspectral techniques yields high accuracy, it also has the disadvantages of requiring expensive acquisition devices and large volumes of image data, a slow acquisition speed and susceptibility to environmental influences, making it difficult to satisfy the demand for a large-scale, low-cost quality inspection method.

Instance segmentation [17] based on deep learning technology [18] can quickly and accurately solve complex problems and realize large-scale image data processing, so it has become one of the most popular research directions in the field of computer vision. Instance segmentation is widely used in many fields, such as industry, transportation, remote sensing images and agriculture. For example, in 2022, in the industrial field, Antwi-Bekoe et al. [19] established an instance segmentation network for the segmentation of industrial insulator defects by using the interactions of cross-latitude information, and the segmentation accuracy achieved for insulator defects reached 89.4%. In the field of transportation, de Carvalho et al. [20] generated a vehicle dataset via semisupervised iterative learning and proposed a spear box-free instance segmentation method, which achieved 90% accuracy with the generated dataset. In 2023, Chen et al. [21] proposed a large-scale building extraction framework in the field of remote sensing images based on super-resolution and instance segmentation to perform instance segmentation for large-scale buildings in cities, and the building segmentation accuracy achieved for satellite images reached 84%. In the agricultural field, Borrenpohl et al. [22] used instance segmentation techniques to segment cherry trees and their dormant fruits through images with active illumination and natural illumination, and the image segmentation accuracy of this approach reached 94%. The applications of instance segmentation in various fields have significant advantages, including low costs, high speeds, high accuracy rates and the capacity for large-scale intelligent quality inspection. Despite the above advantages of instance segmentation, however, a rice seed instance segmentation method has not been reported, mainly because of the similar characteristics, small particles and dense distributions of rice seeds, which make it difficult to accurately detect their quality and segment them.

The existing instance segmentation methods include Mask-R-CNN [23], MNC [24], FCIS [25], RetinaMask [26], SOLO2 [27], Boxinst [28], RTMDet [29], Condinst [30], etc. Mask-R-CNN is very influential in instance segmentation. Based on Faster-R-CNN [31], Mask-R-CNN adds a new mask branch in the region of interest (ROI), and its classification branch and box branch are processed in parallel. Each mask branch of the ROI is a full convolution structure, and the mask is predicted in pixels. In another instance segmentation method, the HTC [32] method, which was inspired by Mask-R-CNN and Cascade-R-CNN [33], a new cascade structure was designed on the basis of Cascade-R-CNN; this structure fully interacts with the feature map information, crosses the mask prediction with the box prediction and introduces a mask path to a newly added semantic segmentation branch. The semantic segmentation branch is integrated with the mask branch to obtain richer contextual information and to supplement the feature information of the existing mask branch. These methods improve the accuracy of target segmentation, but their inference speeds are still slow.

The Yolact [34] method divides the instance segmentation task into two subtasks for parallel processing, which greatly improves the inference speed of the model, but its segmentation accuracy for small targets is general and not specific. Due to the different sizes of the targets, the detection difficulty varies, and according to the definition of the COCO dataset [35], target pixel areas less than 32^2^ are defined as small targets, target pixel areas greater than 32^2^ and less than 96^2^ are defined as medium targets and target pixel areas greater than 96^2^ are defined as large targets. The above methods have been used to conduct much research related to the segmentation accuracy and inference speed achieved for targets and have improved the segmentation accuracy that can be attained with large and medium-sized targets; however, the instance segmentation effects produced for small targets, such as rice seeds, are worse, mainly because rice seeds have dense distributions, small particles and similar physical characteristics, making them difficult to distinguish.

To address the problem of precisely segmenting small rice seed targets in nondestructive rice inspections, a network called the Swin Yolact network (SY-net) is proposed for rice seed instance segmentation that adopts the backbone of Swin’s architecture and the idea of parallelizing tasks based on Yolact. SY-net is built with four main modules to enhance its rice seed detection and segmentation effects. These four modules include a feature extraction module, a feature pyramid fusion module, a prediction head module and a prototype mask generation module. SY-net’s ability to learn the features of rice seeds is enhanced by the use of a Swin-T [36] structure with a self-attentive mechanism [37,38] as a feature extractor in the feature extraction module. In the feature pyramid fusion module, a six-layer feature pyramid network is employed to fuse the feature maps at different stages, and a smaller detection scale is used with the feature maps to enhance the ability of the network to detect rice seeds. Furthermore, a parallel prediction head structure consisting of fully convolutional layers is used in the prediction head module to make full use of the feature maps output by each convolutional layer. A larger feature map is used in the prototype mask generation module to generate the prototype mask. The composition of this network structure is introduced in detail in the Section 2; the training strategy, experimental results and analyses are introduced in the Section 3; and the effectiveness of the method in rice seed segmentation tasks is summarized in the Section 4 of this paper.

## 2. Materials and Methods

**Figure 1:**
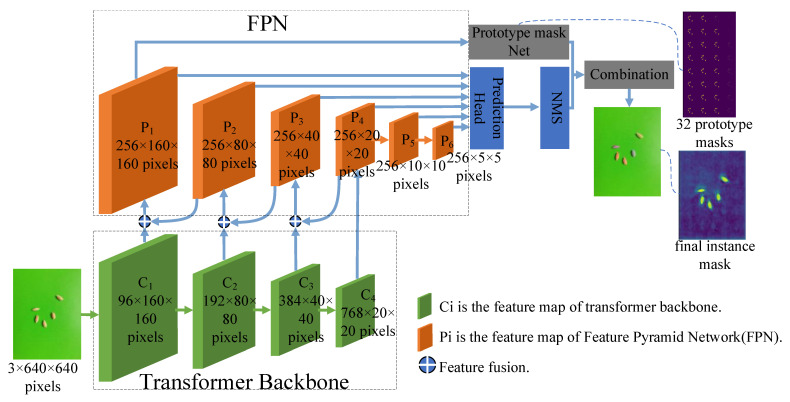
The architecture of SY-net.

The overall architecture of SY-net, shown in Figure 1, consists of six parts; namely, a transformer backbone, a feature pyramid network (FPN), a prediction head, a prototype mask net, a nonmaximum suppression (NMS) mechanism and a combination module. The transformer backbone is the feature extractor module, and the FPN is the feature pyramid fusion module. The prediction head and prototype mask network are the prediction head module and the prototype mask generation module, respectively, both of which are composed of fully convolutional modules. C1, C2, C3 and C4 are the feature map outputs of the transformer backbone at four stages (stages one to four), with sizes of 96 × 160 × 160 pixels, 192 × 80 × 80 pixels, 384 × 40 × 40 pixels and 768 × 20 × 20 pixels, respectively. P1–P6 are the feature map outputs of the FPN at six stages, and P4 comes from C4 and is downsampled to generate P5 and P6. P4 is upsampled and fused with C3 (this is the ⊕ operation in the figure, representing the feature fusion operation) to generate P3, P3 is upsampled and fused with C2 to generate P2 and P2 is upsampled and fused with C1 to generate P1. The sizes of P1–P6, which are the input feature maps of the prediction head, are 256 × 160 × 160 pixels, 256 × 80 × 80 pixels, 256 × 40 × 40 pixels, 256 × 20 × 20 pixels, 256 × 10 × 10 pixels and 256 × 5 × 5 pixels, and P1 is the input feature map of the prototype mask network. The prediction head outputs the prediction value, and the prototype mask network outputs the prototype mask. The NMS [39] module filters the predicted values generated by the prediction head. The combination module combines the outputs of the prediction head and prototype mask network in a linear manner to generate the final instance mask. Figure 1 shows the 32 prototype masks generated by the prototype mask generation network and the mask for the final instance obtained after the combination module.

### 2.1. Dataset

**Figure 2:**
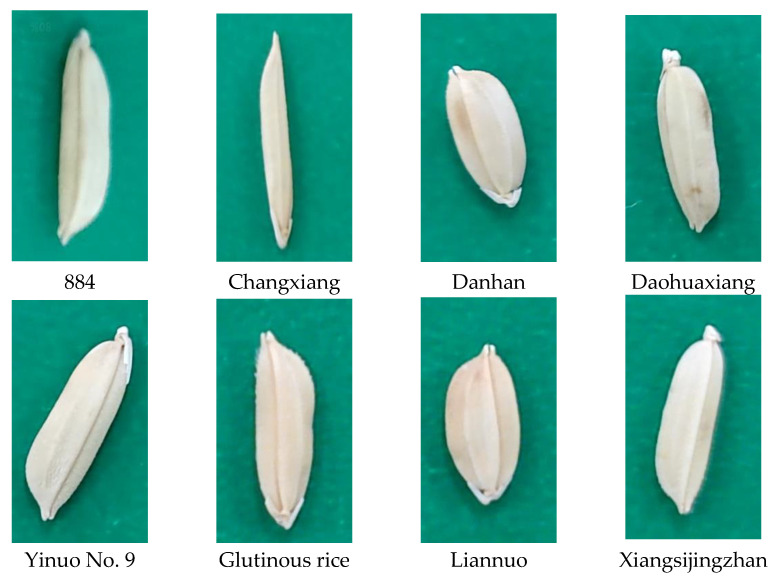
Images of eight rice samples.

In this study, one private rice seed segmentation dataset and two public datasets (Pascal SBD [40] and MS COCO2017) were used to verify SY-net’s instance segmentation capability for small targets. The private rice seed segmentation dataset contains eight categories and 2263 images. As shown in Figure 2, the categories of rice are 884, Changxiang, Danhan, Daohuaxiang, Yinuo No. 9, glutinous, Liannuo and Xiangsijingzhan rice. Utilizing a mobile phone camera (Honour LLD-AL10, 1300 resolution; Shenzhen Zhixin New Information Technology Co., Ltd., Shenzhen, China), the lighting conditions and image capture settings (i.e., the background, focal length, capture angle and distance from the camera to the sample) were set to be random. A ratio of 0.9:0.1 was used to divide the training set and the validation set. The Pascal SBD dataset contains 20 categories with 11,000 images, and MS COCO2017 contains 80 categories with 123,000 images.

### 2.2. Feature Extraction Module

**Figure 3:**
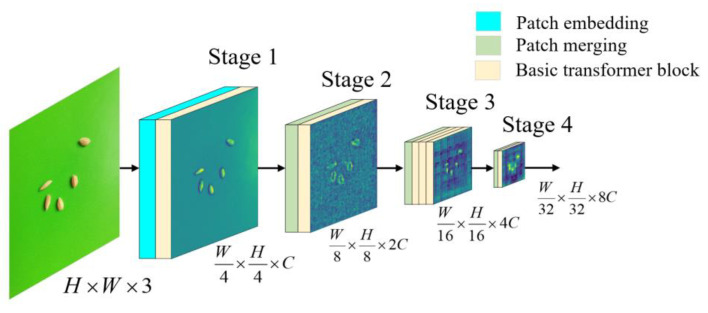
Architecture of the transformer backbone. *H* and *W* are the height and width of the feature map, respectively, and *C* is the number of channels in the feature map.

The physical features of rice seeds are similar, and their details are not obvious. It is difficult for existing feature extractors to focus on the key features of rice seeds. In this study, a feature extractor was built by using the transformer architecture. The learning of the physical features of small targets such as rice seeds can be enhanced through an attention mechanism, and the detailed process of extracting features from small targets can be improved from different angles. The network structure diagram for feature extraction is the transformer backbone, as shown in Figure 3. In this figure, the transformer backbone consists of three large modules; namely, a patch embedding module (bright cyan cuboid), a patch merging module (light green cuboid) and a basic transformer block (light yellow cuboid). Stages 1–4 correspond to C1–C4 in Figure 1, respectively.

In feature extraction networks, the features of small targets gradually disappear via continuous convolution, and the existing mainstream feature extractors are not good at extracting the detailed physical features of small targets, such as rice seeds. In this paper, a transformer-based feature extraction network with an attention mechanism is introduced that can learn the texture, colour and edge information for rice seeds to enhance the extraction of detailed physical rice seed feature information. SY-net constructs a transformer backbone through the superposition of the three main modules to extract and transfer target feature information. Compared with existing mainstream feature extraction networks, such as VGG [41] ResNet50, ResNet101 [42], Darknet [43], DenseNet [44] and MobileNetV3 [45], the feature extractor can generate feature maps with richer and more effective feature information.

#### 2.2.1. Patch Embedding

The patch embedding module (bright cyan cuboid in Figure 3) is used to adjust the width, height and number of channels of the input image. Patch embedding downsamples the input image by a factor of 4 and outputs a specified channel *C*.

**Figure 4:**
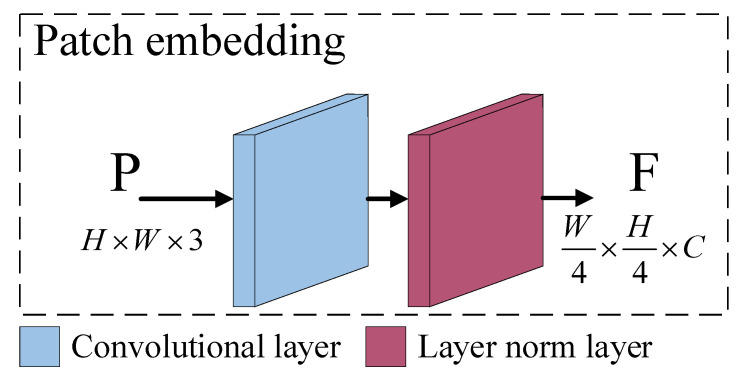
Architecture of the patch embedding module. P is the input image, and F is the output feature map.

**Figure 5:**
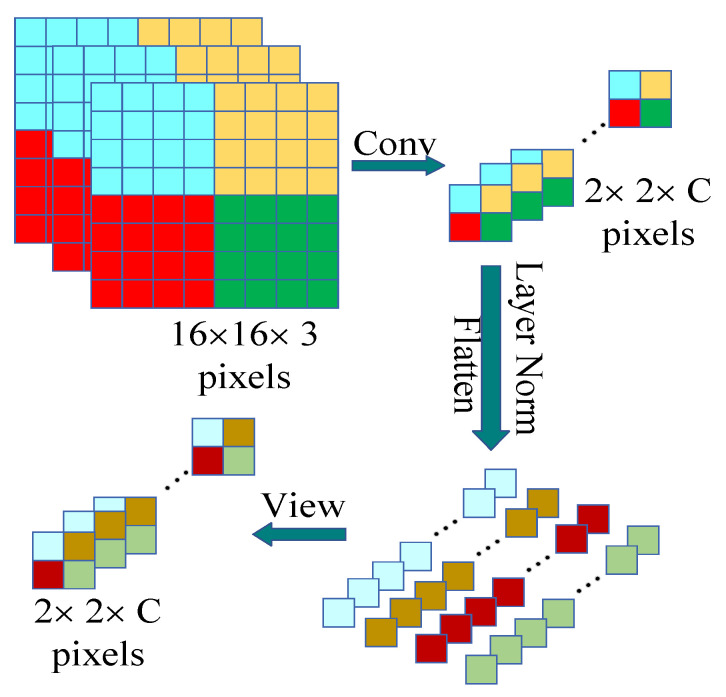
Process flow of patch embedding. Conv is the convolutional process, and Layer Norm is the layer normalization process. *C* is the number of channels in the feature map. The flattening layer flattens the feature map. Viewing is used to restore the flattened data to a feature map.

As shown in Figure 4, the patch embedding module is composed of a convolutional layer and a layer normalization layer. The input image is downsampled by using a convolutional layer with a kernel size of 4 × 4 and a span of 4, and a feature map with the specified number of channels (depth) is output, flattening the height and width of the feature map. Then, the layer normalization layer is used to normalize the map in the depth direction and, finally, the feature map is restored through the viewing method. The specific process is shown in Figure 5.

In Figure 5, the image size used as an example is 16 × 16 × 3 pixels. First, the input image is downsampled to generate a feature map 2 × 2 × *C* pixels in size; this is followed by flattening and layer normalization processing in the depth direction of the generated feature map. Finally, the processed data are restored to a feature map 2 × 2 × *C* pixels in size by the viewing method as the output feature map.

#### 2.2.2. Patch Merging

The patch merging module (light green cuboid in Figure 3) is used to adjust the widths, heights and numbers of channels for the input feature maps at different stages. Patch merging downsamples the input feature maps by a factor of 2, and the number of output channels is double the number of input channels.

**Figure 6:**
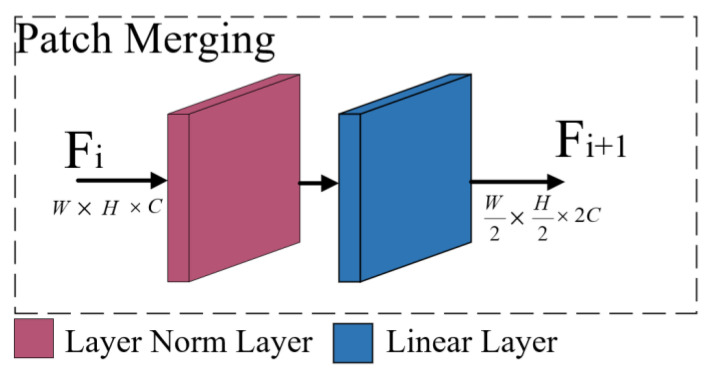
Architecture of the patch merging module. F_i_ is the input feature map, and F_i+1_ is the output feature map.

**Figure 7:**
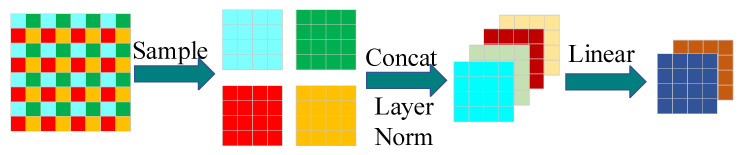
Process flow for the patch merging module. The sampling function is used to sample the feature map. Concat concatenates the feature map in the depth direction. Layer Norm is the layer normalization process. Linear is a fully connected layer that performs linear mapping.

As shown in Figure 6, the patch merging module is composed of a layer normalization layer and a linear layer. First, the patch merging module performs equally spaced sampling on the feature maps, and the four feature maps generated by sampling are concatenated in the depth direction and processed by the layer normalization layer. Finally, they are mapped in the depth direction through the linear layer. The specific process is shown in Figure 7.

In Figure 7, a feature map 8 × 8 pixels in size is used as an example. First, the feature map is sampled at equal intervals (the sampling interval is 2) to generate four feature maps 4 × 4 pixels in size. Then, the four generated feature maps are connected in the depth direction. Next, layer normalization processing is performed, and linear mapping is executed by the linear layer to generate two feature maps with dimensions of 4 × 4 pixels as the output feature maps.

#### 2.2.3. Basic Transformer Block

**Figure 8:**
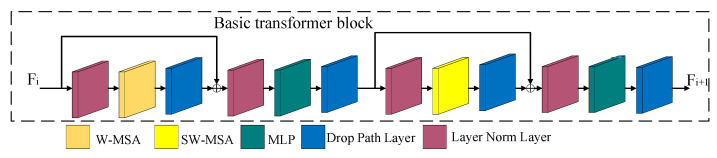
The architecture of the basic transformer block. F_i_ is the input feature map, and F_i+1_ is the output feature map.

The basic transformer block (light yellow cuboid in Figure 3) is used to extract the feature information from the feature map. As shown in Figure 8, the basic transformer block consists of a window multi-head self-attention (W-MSA) mechanism, a shift window multi-head self-attention (SW-MSA) mechanism, a multilayer perceptron (MLP), a path dropping layer and a layer normalization layer. The W-MSA and SW-MSA mechanisms are the core of the basic transformer block.

Compared to a traditional convolutional neural network, the ability of the present network to use convolution to extract small-target features is weak. Multi-head self-attention (MSA) is used to extract the feature map information, as it makes it possible to learn the features in more detail and can generate high-quality feature maps. Two MSA modules, W-MSA and SW-MSA, are used in the basic transformer block to strengthen its ability to extract detail features from small targets. MSA functions based on self-attention (SA), which is calculated as shown in Formula (1). In Formula (1), *Attention (Q,K,V)* is the feature output of the SA, and *Q*, *K* and *V* are obtained through the operation of the input vector and the trainable parameter matrices *W^q^, W^k^* and *W^v^*. The calculation formula is shown in Formula (2), where *X* is the input feature vector, d is the dimensionality of *K* and *B* is the bias.

(1) $$Attention\left(Q,K,V\right)=soft\max(\frac{QK^{T}}{\sqrt{d_{k}}}+B)V$$

(2) $$\left(\begin{array}{c} Q\\ K\\ V \end{array}\right)=X_{}\;\times \left(\begin{array}{c} W^{q}\\ W^{k}\\ W^{v} \end{array}\right)$$

MSA splits the calculated *Q*, *K* and *V* according to the number of heads and combines *Q*, *K* and *V* after splitting to form the head. The calculation formula of MSA is shown in Formula (3).

(3) $$MutiHead\left(Q,K,V\right)=Concat\left(Head_{1},Head_{2},\ldots Head_{n}\right)W^{o}\;where\begin{array}{cc} & Head_{i} \end{array}=Attention\left(QW_{i}{}^{Q},KW_{i}^{K},VW_{i}^{V}\right)$$

In Formula (3), *MutiHead(Q,K,V)* is the feature output of the MSA. *W_i_^Q^, W_i_^K^* and *W_i_^V^* are used to split *Q*, *K* and *V*, respectively, and then the SA operation is performed. Then, each head (output of the SA) is spliced, and *W^o^* is used to fuse the spliced feature map.

W-MSA divides the input feature map into four windows of the same size, and MSA feature extraction is conducted for each window. SW-MSA is a more detailed window division process based on W-MSA. The feature map is divided into nine windows, and MSA feature extraction is conducted. As shown in Figure 8, the basic transformer block module in the transformer backbone uses W-MSA and SW-MSA alternately to realize feature learning and information exchange for the notable features of small targets and to build the global feature relationship.

### 2.3. Feature Pyramid Fusion Module

When the network extracts features from the feature map, the downsampling of the target loses important spatial features, resulting in difficulties when attempting to detect small targets and the inability to obtain accurate segmentation results. Therefore, in this study, the existing feature pyramid network [46] was improved by using smaller detection scales on larger feature maps to resolve these detection difficulties. A six-layer feature pyramid network was designed to perform feature fusion on the output feature maps of the transformer backbone at different stages and to address the problem of spatial information loss. The feature fusion module makes full use of the feature map information contained in each feature map by performing up- and downsampling and feature fusion on the feature maps output by the feature extraction module at different stages, thus improving the mask segmentation capability of the network for small targets such as rice seeds. Four-stage feature maps obtained from the transformer backbone are used as the inputs of the feature pyramid fusion module, and six-stage feature maps are output.

The structure of the FPN is shown in Figure 1. P4 is derived from C4; P4 is downsampled to generate P5 and P6. P4 is upsampled and fused with C3, C2 and C1 to generate P3, P2 and P1 in turn. Compared with the existing feature fusion network, the proposed FPN can generate larger feature maps with more abundant feature information. At the same time, six different scales are used for prediction, and three different proportions are used to adjust the sizes of the six FPN feature maps. The sizes of the prediction scales are [12, 24, 48, 96, 192, 384], and the proportions are [1, 1/2, 2/1]. In a larger feature map, a smaller prediction scale is used to strengthen the detection ability of the network for small targets. Three different proportions can make the prediction and positioning results more accurate.

### 2.4. Prediction Head Module

**Figure 9:**
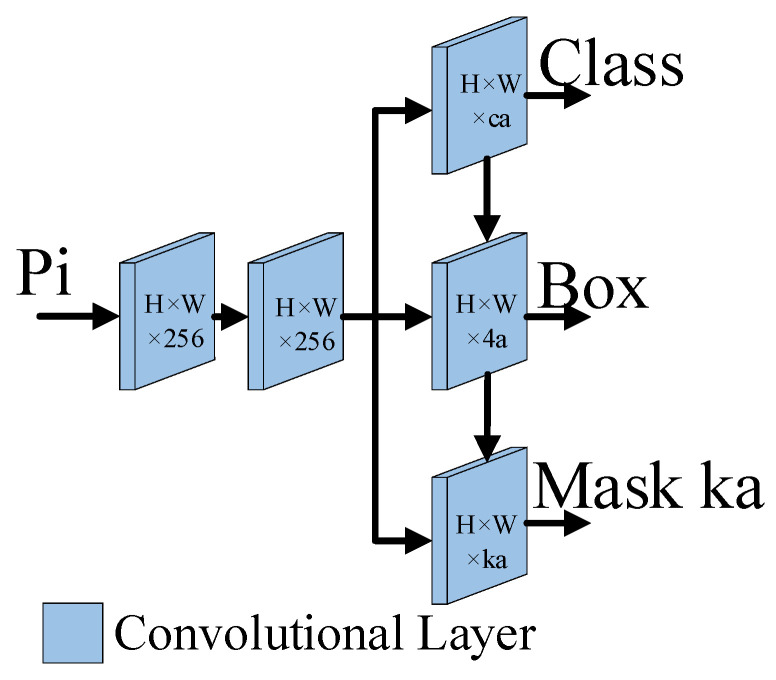
The architecture of the prediction head network. Pi is the feature map of the FPN. ca, 4a and ka are the predicted categories, coordinates and mask coefficients, respectively.

Many existing prediction heads predict categories and regression boxes independently and do not make full use of the predicted feature map output. In this paper, a parallel branch network is used, and the feature map output by each branch is fused with the features of the other branches. The prediction head adopts a parallel branch structure and a weight-sharing mechanism. This method has fewer weight parameters and a faster detection speed than other complex prediction heads. The input of the prediction head comes from the feature maps of the FPN, and it predicts all the feature maps generated by the FPN. In Figure 1, the prediction head is the prediction head module, and the branch structure diagram of the network is shown in Figure 9.

As shown in Figure 9, two convolutional layers extract features from the input feature maps, and the other three convolutional layers are parallel branches that share the feature maps output from the first two convolutional layers; the latter layers predict the class, box and mask coefficients ka, respectively. The feature maps generated by each convolutional layer are fully utilized, and the feature maps generated by the convolutional layer for the category output are fused with the feature maps for predicting a regression box. Then, the feature maps generated by the convolutional layer for predicting the regression box and the feature maps for generating the mask coefficients are fused to predict the mask coefficients. Through such a feature fusion process, the feature output of each convolutional layer can be utilized and a more refined prediction result can be produced.

In this paper, the loss calculation for the class of a given target uses the softmax cross-entropy function to train with *c* + 1 samples (*c* positive labels and 1 background label), and the ratio for the positive and negative samples selected for training is 3:1. The smooth *L*_1_ loss function is used to train the coordinate regression function of the prediction box because the smooth *L*_1_ function has the advantages of good robustness, insensitivity to outliers and small gradient changes. The smooth *L*_1_ loss function is shown in Formula (4).

(4) $$L_{loc}\left(t^{u},v\right)=\underset{i\in \left\{x,y,w,h\right\}}{\sum }smooth_{L_{1}}\left(t_{i}^{u}-v_{i}\right)$$

where *t^u^* is the coordinate predicted by the bounding-box regressor and *v* is the bounding-box coordinate of the true label.

(5) $$smooth_{L_{1}}=\left\{\begin{array}{l} 0.5x^{2}\\ \left|x\right|-0.5 \end{array}\right.\begin{array}{c} \;if\;\begin{array}{cc} x<1 & \end{array}\\ otherwise \end{array}$$

In Formula (5), *x* is the difference between the true label and the predicted label.

### 2.5. Prototype Mask Generation Module

**Figure 10:**
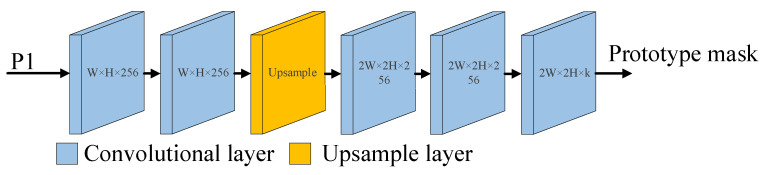
The architecture of the prototype mask network. P1 is the largest and deepest feature map of the FPN. k is the number of prototype masks generated.

In the instance segmentation task, the mask generation network usually adopts a fully convolutional network, and the quality of the mask is closely related to the size of the feature map. Therefore, in this paper, a prototype mask generation network composed of five convolutional layers is used to double the height and width of the input feature map, and this larger feature map is used to generate the mask. The input of the prototype mask generation module is P1 in the FPN. This is because the masks generated by deep-level feature maps in deep neural networks have better robustness, and larger feature maps contain more semantic information, enabling the generation of masks with higher quality. Moreover, this approach has better mask generation performance for small targets. P1 is the largest and deepest feature map in the FPN. By upsampling P4, P3 and P2 in turn, the smallest feature map is enlarged and then fused with the features of the next layer so that the bottom feature map has more abundant feature information; this improves the segmentation performance achieved for rice seeds. In Figure 1, the prototype mask network is the prototype mask generation module, and the branch structure diagram of the network is shown in Figure 10.

As shown in Figure 10, for the convolution in the fully convolutional network, first, two convolutional layers are used. The number of input channels is 256, the number of output channels is 256, the convolution kernel size is 3 × 3, the stride is 1 and the padding is 1. Then, the height and width of the feature map are doubled through an upsampling layer. The feature map passes through two more convolutional layers (with the same parameters as the previous convolutional layer) and finally passes through a convolutional layer with 256 input channels, k output channels, a kernel size of 1 × 1, a stride of 1 and padding of 0, where k is the number of prototype masks generated. The rectified linear unit (ReLU) activation function is used to activate the prototype mask because it is a nonlinear function that has the advantages of simple operation, fast convergence and not having a saturation region. By using the ReLU function, the inference speed of the network can be accelerated, and the problem of gradient disappearance can be avoided. After a series of convolution and upsampling operations, the width and height of the output feature map will be double those of the input feature map. Performing feature extraction through larger feature maps can produce finer masks and richer semantic information.

### 2.6. NMS Processing

During the inference process implemented by the network, many candidates are generated for the regression bounding boxes and the class confidence. The existing method adopts traditional NMS processing, which filters the candidate boxes below the target confidence level while retaining the candidate boxes with confidence levels above the threshold. Although the traditional NMS approach can effectively deal with the candidate boxes, the shortcoming of its calculation process is that the candidate boxes are sorted according to their confidence scores. Then, the candidate boxes are deleted, which can cause the calculations to be executed in sequence, making this approach very slow. To improve the reasoning speed of the network, Fast NMS is used in this paper. Fast NMS is an NMS algorithm that can quickly conduct filtering. It performs parallel matrix calculations on the basis of traditional NMS. It first obtains the top *n* detection targets in descending order of confidence for each class and then calculates the intersection over union (IOU) values among these *n* detection targets to obtain an IOU matrix with a size of *c* × *n* × *n*, where *c* is the number of categories and *n* × *n* is a diagonal and symmetrical matrix. Second, the values of the lower triangle and the diagonal of the diagonal matrix are set to 0 (*X_kij_* = 0, ∀*k*, *j*, *i* ≥ *j*), and then the maximum value of each column is obtained, as shown in Formula (6). Finally, the candidate boxes with high overlap and low confidence rates that are higher than a certain threshold are filtered out, and the best candidate boxes for each category are screened out. In Formula (6), *k* represents the *k*th category, *i* and *j* represent the index of the IOU matrix of that category, *X_kij_* is the upper triangular matrix of the *k*th category and *K_kj_* is the best regression box of that category, respectively.

(6) $$K_{kj}=\underset{i}{max}\left(X_{kij}\right)\begin{array}{cc} & \forall k,j \end{array}$$

### 2.7. Combination Module

To generate the final instance mask, the outputs of the prototype mask generation module and the prediction header module are linearly combined, and then the nonlinear sigmoid activation function is used for activation to generate the final mask. The linear combination of the outputs of the two modules can be realized by using a matrix. The calculation process is shown in Formula (7):

(7) $$M=\sigma \left(PC^{T}\right)$$

where *P* is the matrix of the prototype mask of *h* × *w* × *k* and *C* is the matrix of the mask coefficient of size *n* × *k* (*n* is the candidate target screened after NMS processing). *σ* denotes activation processing. *T* stands for the transpose of the matrix. This simple linear combination has the advantage of being very fast.

## 3. Results

In this paper, a single RTX-3090 GPU with 24 GB of video memory was used to train the network model. The design of the network was based on a basic environment with Python 3.8 and PyTorch 1.10. Random clipping, random mirroring, random flipping and other data enhancement processes were used to expand the dataset and to strengthen the training process for the network model. The trained weight of the transformer backbone was adopted and transferred to the feature extractor of SY-net. The batch size was set to 8, the weighted adaptive moment estimation (AdamW) optimizer was adopted to optimize the network, the weight decay rate was set to 5 × 10^−4^ and the initial learning rate was set to 1 × 10^−4^. For the rice seed segmentation dataset, 80,000 iterations of training (120,000 iterations for the Pascal SBD dataset and 800,000 iterations for the COCO2017 dataset) and 500 iterations of warm-up training were utilized; the learning rate for the warm-up training process was set as 1 × 10^−5^, and the NMS processing threshold was set as 0.5. The input image resolution was 640 × 640 pixels.

### 3.1. Experimental Verification Conducted with the Rice Seed Segmentation Dataset

To verify the effectiveness of the designed module in the rice seed segmentation scenario, ablation experiments were conducted with the rice seed segmentation dataset. As shown in Table 1, the designed modules improved the segmentation accuracy of the rice seed mask by 7.27%, 0.39% and 0.8%, proving the effectiveness of the designed modules. The feature fusion module improved the accuracy the most (by 7.27%), which indicates that utilizing smaller detection scales with feature maps with rich feature information can enhance a network’s ability to detect small targets. The prediction head module and the prototype mask generation module improved the segmentation accuracy for rice seeds by 0.39% and 0.8%, respectively, indicating that these two modules can make better use of the feature information from the feature map. 

To verify the improvements of the designed method in the rice seed segmentation scenario, comparative experiments were conducted with the rice seed segmentation dataset, and the results are shown in Table 2. In Table 2, AP_50_ and AP_75_ represent the average precision levels of the mask when the IOU was 0.5 and 0.75, respectively. The models shown in Table 2 were all trained with 80,000 iterations. According to the experimental results, SY-net had the highest mAP with the rice seed segmentation dataset, reaching 90.71%, followed by Condinst and Boxinst, which reached 85.20% and 82.90%, respectively. It can be seen that some of the models in Table 2 had similar or the same accuracy values for AP_50_ and AP_75_, which was because the mAP was the mean value for the AP of the IOU thresholds in the range of 0.5–0.95. When a network converges, with a higher mAP value, the AP accuracy of the neighbouring IOU thresholds will gradually converge to the same value.

**Figure 11:**
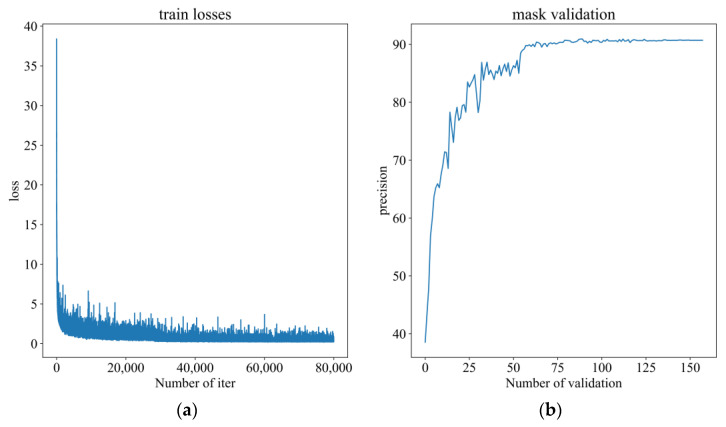
Total loss curve and segmentation precision curve. (**a**) Total loss curve with the number of iterations. (**b**) the segmentation precision curve with the number of validations.

With the rice seed segmentation dataset, SY-net used 80,000 training iterations (314 epochs) and 158 validation iterations, and the total training loss and validation precision are shown in Figure 11. Figure 11a shows the total loss curve produced during training, where the abscissa is the number of iterations and the ordinate is the loss value. Figure 11b show the segmentation precision curve produced during validation, where the abscissa is the number of validations and the ordinate is the validation precision. According to the data in Figure 11, the loss function gradually converged at the 40,000th iteration, and the validation precision tended to be stable after the 80th validation.

**Figure 12:**
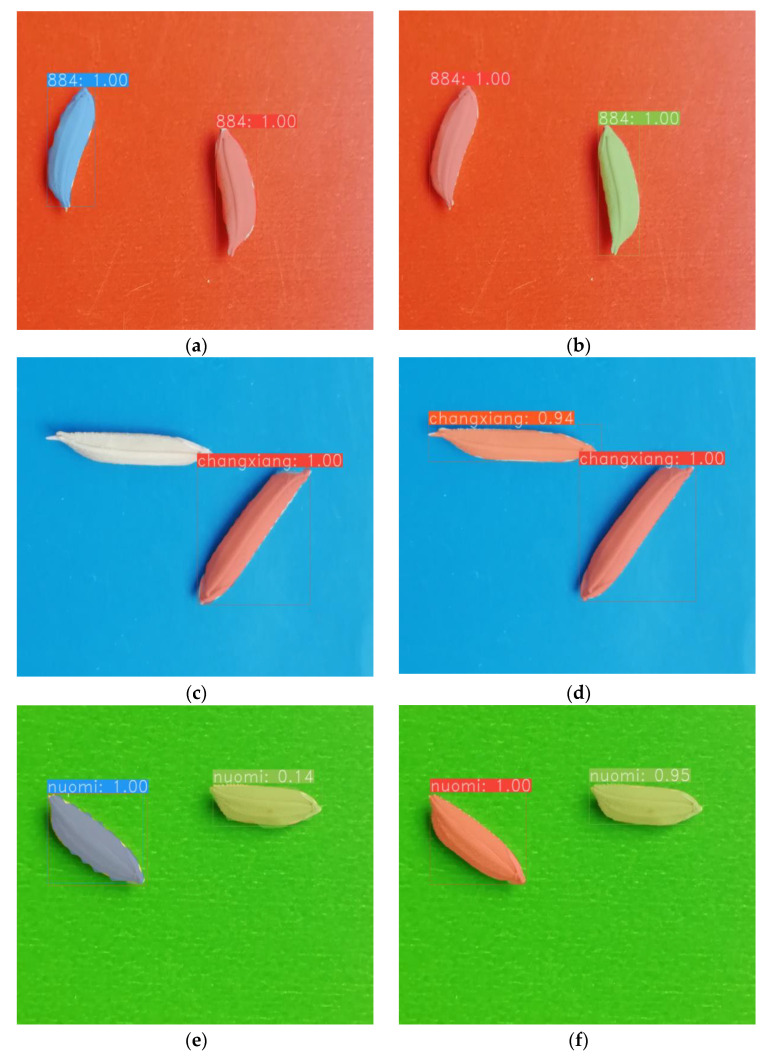

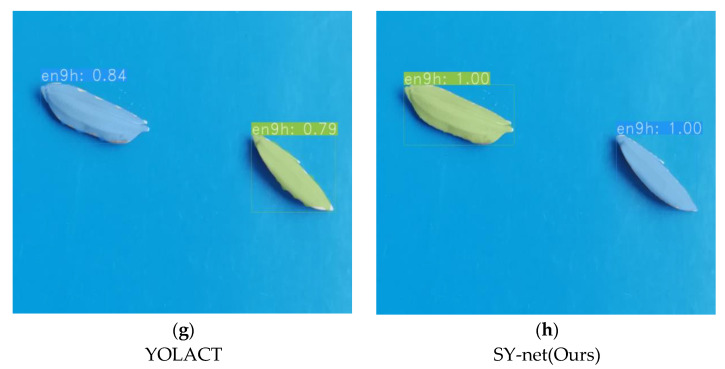
The comparison of segmentation effect between the proposed method and YOLCAT with the rice seed segmentation dataset. (**a**,**c**,**e**,**g**) are the segmentation effects of YOLCAT on different varieties of rice seed targets. (**b**,**d**,**f**,**h**) are the segmentation effects of SY-net on the corresponding varieties of rice seed targets.

The experimental results show that the proposed method has high precision for small targets such as rice seeds. Figure 12 shows the segmentation effect achieved by the proposed method for rice seeds.

Figure 12a,c,e,g show the segmentation effect of YOLACT, and Figure 12b,d,f,h show the segmentation effect of SY-net. For the same rice seed data, based on the comparison images, it can be seen that SY-net yielded better detection and segmentation effects, and the generated rice seed mask was more detailed and complete. The method described in this paper is more capable of learning the detailed features of rice seeds and has better performance in terms of segmenting the instances of rice seeds.

### 3.2. Comparison Experiments with the Pascal SBD and COCO2017 Datasets

To verify that the proposed method does not degrade the accuracy for medium and large targets while targeting small targets such as rice seeds, comparative experiments were conducted with the Pascal SBD dataset and the COCO2017 dataset.

The proposed method was first validated with the Pascal SBD dataset. Table 3 shows the mask evaluation results obtained with the method in this paper with the Pascal SBD dataset. The precision of the method in this paper reached 76.4% and 63.0% for the AP_50_ and AP_70_ metrics, respectively. The accuracies of MNC-VGG16 were 63.5% for AP_50_ and 41.5% for AP_70_. The accuracies of FCIS-R101-C5 were 65.7% for AP_50_ and 52.1% for AP_70_. The accuracies of YOLACT-R50 were 72.3% for AP_50_ and 56.2% for AP_70_. Compared with YOLACT-R50, SY-net improved the AP_50_ and AP_70_ by 4.1% and 6.8% respectively, but the FPS was much slower. There are two reasons for this. First, SY-net uses two self-attention mechanism modules in the backbone network; namely, W-MSA and SW-MSA. Compared with a convolutional module, self-attention mechanism modules have greater computational complexity. Second, SY-net uses a smaller detection scale, which increases the amount of computation. As shown by the data in Table 3, the method described in this paper achieved the best performance for both AP_50_ and AP_70,_ indicating that the proposed method could also obtain good segmentation accuracy for the targets of different sizes in the Pascal SBD dataset.

To further validate the segmentation ability of the proposed method for targets of different sizes, the performance of SY-net was evaluated with the COCO2017 validation set in terms of six aspects: mAP, AP_50_, AP_75_, AP_S_, AP_M_ and AP_L_ (where AP_50_ and AP_75_ are the AP achieved when the IOU thresholds were 0.5 and 0.75, respectively, and AP_S_, AP_L_ and AP_M_ are the AP achieved when segmenting small, medium and large targets, respectively.) The results of the mask evaluation performance comparison conducted for SY-net and the mainstream algorithms with the COCO2017 dataset are shown in Table 4. From the data in Table 4, it can be seen that SY-net performed best in terms of AP_L_ and AP_S_, with precision levels of 52.7% and 16.5%, respectively. RetinaMask performed best in terms of mAP, with a precision value of 36.6%. Mask-R-CNN had the best performance with respect to AP_50_, AP_75_ and AP_M_, with accuracies of 58.0%, 37.5% and 38.1%, respectively. In general, SY-net did not show worse segmentation capability with medium and large targets in the COCO dataset, and it was faster at the same level of accuracy.

**Figure 13:**
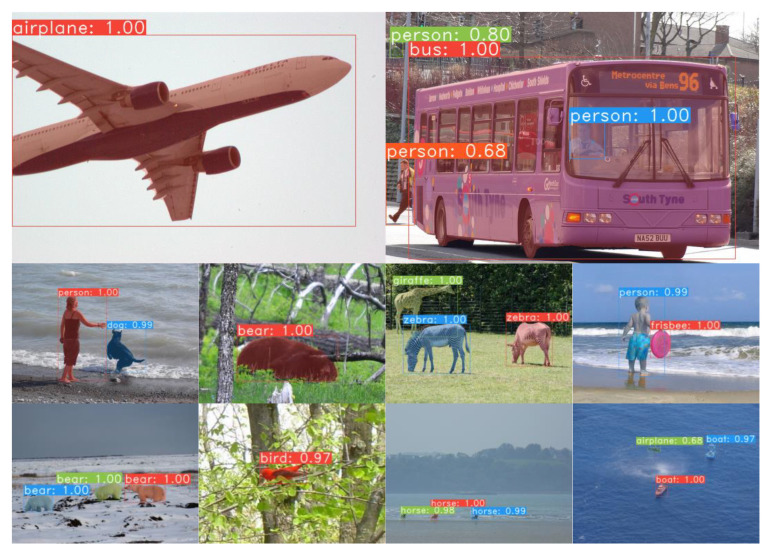

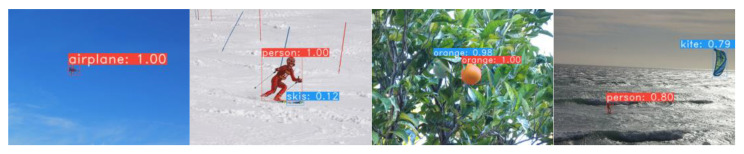
Mask segmentation results obtained by SY-net on the COCO2017 dataset.

Figure 13 displays the detection and segmentation performance achieved by SY-net with the COCO2017 dataset. The first row shows the segmentation effect with large targets, the second row shows the segmentation effect with medium targets, and the third and fourth rows show the segmentation effect with small targets. Combining Table 4 and Figure 13, it can be seen that SY-net also showed good segmentation with the COCO2017 dataset for targets of different sizes.

## 4. Conclusions

In this paper, a very effective rice seed segmentation network, SY-net, was proposed. The ability of the network to learn detailed rice seed features was enhanced by using the transformer backbone in the feature extraction module. A six-layer feature fusion module was designed to fuse the feature maps obtained at different stages and use smaller scales for seed detection. A parallel prediction head module was designed to fully utilize each predicted feature map to acquire more accurate predictions. A larger feature map was used in the prototype mask generation module to generate higher-quality prototype masks. As a result of the above improvements and design, the method achieved 90.71% accuracy for mask segmentation with a rice seed segmentation dataset and an accuracy of 16.5% for small target segmentation with COCO2017. The developed network improves the efficiency of rice seed segmentation and has good application prospects in relation to rice seed quality inspection. In future research, the research objective can be further extended to the segmentation of other grains, and more precise instance segmentation can be realized in different grain quality inspection environments, which can provide further contributions to the grain industry.

## Figures and Tables

**Table 1:** Results of ablation experiments on SY-net with the seed segmentation dataset.

Method	Feature Extraction Module	Feature Pyramid Fusion Module	Prediction Head Module	Prototype Mask Generation Module	mAP
SY-net	√				82.25%
	√	√			89.52 (+7.27)%
	√	√	√		89.91 (+0.39)%
	√	√	√	√	90.71 (+0.8)%

**Table 2:** SY-net mask evaluation results obtained with the rice seed segmentation dataset.

Method	mAP	AP_50_	AP_75_
SY-net	90.71%	98.58%	98.58%
Yolact	80.04%	93.27%	93.27%
SOLO2	75.40%	90.00%	83.50%
Boxinst	82.90%	96.70%	96.40%
RTMDet	81.80%	97.60%	97.30%
Condisnt	85.20%	93.90%	93.90%

**Table 3:** SY-net mask evaluation results obtained with the Pascal SBD dataset.

Method	FPS	AP_50_	AP_70_
MNC-VGG16	2.8	63.5%	41.5%
FCIS-R101-C5	9.6	65.7%	52.1%
YOLACT-R50	47.6	72.3%	56.2%
SY-net (ours)	20.0	76.4%	63.0%

**Table 4:** SY-net mask evaluation results obtained with the COCO2017 dataset.

Method	FPS	mAP	AP_50_	AP_75_	AP_S_	AP_M_	AP_L_
Mask-R-CNN	8.6	35.7%	58.0%	37.8	15.5%	38.1%	52.4%
FCIS	6.0	29.5%	51.5%	30.2%	8.0%	31.0%	49.7%
RetinaMask	4.7	36.6%	55.4%	36.9%	14.3%	36.7%	50.5%
YOLACT	33.5	29.8%	48.5%	31.2%	9.9%	31.3%	47.7%
SY-net (ours)	16.2	33.7%	55.3%	34.9%	16.5%	36.0%	52.7%

## Data Availability

The data that support the findings of this study are available from the authors upon request.
